# Fast Track Liver Resection: The Effect of a Comprehensive Care Package and Analgesia with Single Dose Intrathecal Morphine with Gabapentin or Continuous Epidural Analgesia

**DOI:** 10.1155/2009/271986

**Published:** 2009-12-15

**Authors:** Jonathan B. Koea, Yatin Young, Kerry Gunn

**Affiliations:** The Departments of Surgery and Anaesthesia, Auckland City Hospital, Auckland 1023, New Zealand

## Abstract

*Background*. A comprehensive care package for patients undergoing hepatectomy was developed with the aim of minimal physiological disturbance in the peri-operative period. Peri-operative analgesia with few gastrointestinal effects and reduced requirement for intravenous (IV) fluid therapy was central to this plan. *Methods*. Data on 100 consecutive patients managed with continuous epidural infusion (*n* = 50; bupivicaine 0.125% and fentanyl 2 *μ*g/mL at 0.1 mL/kg/hr) or intrathecal morphine (*n* = 50; 300 *μ*g in combination with oral gabapentin 1200 mg preoperatively and 400 mg bd postoperatively) was compared. *Results*. The epidural and intrathecal morphine groups were equivalent in terms of patient demographics, procedures and complications. Patients receiving intrathecal morphine received less intra-operative IV fluids (median 1500 mL versus 2200 mL, *P* = .06), less postoperative IV fluids (median 1200 mL versus 4300 mL, *P* = .03) than patients receiving epidural infusion. Patients managed with intrathecal morphine established a normal dietary intake sooner (16 hours versus 20 hours, *P* = .05) and had shorter hospital stays than those managed with epidural infusions (4.7 ± 0.9 days versus 6.8 ± 1.2 days, *P* = .02). *Conclusions*. Single dose intrathecal morphine is a safe and effective means of providing peri-operative analgesia. Patients managed with intrathecal morphine have reduced peri-operative physiological disturbance and return home within a few days of hepatic resection.

## 1. Introduction

Historically, hepatic resection has been associated with significant blood loss [1], and surgical and anesthetic strategies to manage patients undergoing hepatic resection have evolved with the aim of limiting hemorrhage. Surgical techniques have included control of relevant hepatic segmental inflow and outflow structures to vascularly isolate the liver, or part thereof, prior to parenchymal division. Continuous or intermittent mass inflow control (Pringle maneuver) has also been employed during parenchymal division to reduce intraoperative blood loss. Anesthetic strategies have included perioperative intravenous fluid loading to provide a physiological buffer against sudden hypovolemia, and epidural analgesia to reduce central venous and hepatic venous pressure. In addition, these patients have often been cared for in intensive care or high dependency units and restricted to bed because of concern over possible postoperative bleeding.

While these surgical and anesthetic techniques have contributed toward making hepatic resection safer with perioperative mortality rates of under 1% routine in many specialized units, hospital stays remain prolonged ranging from 8 to 14 days [2, 3]. The reasons for hospital stays of this duration are multiple and can reflect the effects of hemorrhagic complications occurring in the perioperative period, later biliary or septic complications, or the development of hepatic insufficiency. Prolonged hospital stay may be partly attributable to delayed mobilization, delay in resuming adequate oral intake, and fluid balance derangements.

We hypothesized that recovery from hepatic resection could be improved by introducing a comprehensive care plan to manage patients in the perioperative period and during subsequent recovery. Central to this care plan was the use of single dose intrathecal morphine rather than continuous epidural analgesia to reduce perioperative intravenous fluid requirements resulting in improved gastrointestinal function, earlier mobilization, and earlier hospital discharge.

## 2. Methods

### 2.1. Demographics and Patient Details

All patients were referred for hepatic resection and were managed at Mercy Hospital in Auckland, New Zealand. Patient details were recorded in a prospective, clinical database. In particular, details were recorded of the patients demographics, ASA grade, diagnosis, procedure, intraoperative blood loss, parenchymal quality, complications, duration of hospital stay, and status at follow up. Subsequently patient charts were reviewed to determine the total intravenous fluid requirement intraoperatively and during their hospital stay, the use of postoperative diuretic therapy, daily body weights, time to first mobilization postoperatively, time to passage of flatus postoperatively, time to resumption and tolerance of normal diet postoperatively, and total daily analgesic requirement. Hepatic resections were described using the Brisbane nomenclature [4]. A major resection comprised resection of 4 or more hepatic segments while a minor resection was defined as resection of less than 4 hepatic segments.

Data on two patient groups were recorded. Fifty consecutive patients undergoing hepatectomy managed with epidural analgesia between January 2005 and December 2005 was compared with 50 consecutive patients undergoing hepatectomy managed with intrathecal morphine and gabapentin between January 2006 and February 2007.

### 2.2. Concomitant Therapy

All patients received a total of 3 doses of perioperative antibiotic (cefoxitin (Mayne Pharma Pty Ltd, Mulgrave, Victoria, Australia) 1 gm Q8 hourly). The first dose was given at induction of anesthesia. Patients undergoing a biliary reconstruction also received further postoperative antibiotic therapy as indicated by the results of bile cultures taken intraoperatively. In addition to antibiotic therapy all patients received 40 mg of omeprazole (Losec, Astra Zeneca Pty Ltd, North Ryde, New South Wales, Australia) daily as ulcer prophylaxis and 5000 units unfractionated heparin (DBL, Mulgrave, Victoria 3170, Australia) subcutaneously twice daily providing the postoperative INR was less than 1.5 (measured daily).

### 2.3. Anaesthesia and Postoperative Analgesia

Patients managed with thoracic epidural anesthesia received a standard continuous infusion of bupivicane 0.125% (Marcain, Astra Zeneca Pty Ltd, North Ryde, New South Wales, Australia) and fentanyl (Astra Zeneca Pty Ltd, North Ryde, New South Wales, Australia) 2 *μ*g/mL at 0.1 mL/kg/hr (up to a maximum of 8 mL/hr) which represented the institutional protocol for epidural infusions. The epidural catheters were inserted immediately pre-operatively and weaned 72 hours postoperatively as this was deemed the optimum time to minimize epidural site infections and to sustain regular oral analgesic administration. At this time the morning dose of subcutaneous heparin dose was withheld and patients received a single dose of 2 mg of morphine into the epidural space prior to the catheters removal. This was supplemented with a dose of oral morphine of 10–20 mg (Douglas Pharmaceuticals, Auckland, New Zealand) and tramadol (Tramal, CSL Ltd, Parkville, Victoria, Australia) of 50–100 mg orally to maintain adequate analgesia after discontinuing the epidural infusion. Patients managed with single dose intrathecal morphine received 300 *μ*g of morphine with oral gabapentin (Neurontin, Pfizer Pharmaceuticals, Auckland, New Zealand) of 1200 mg pre-operatively and 400 mg bid postoperatively) and parecoxib 40 mg IV (Dynastat, Pfizer Pharmaceuticals, Auckland, New Zealand). Both patient groups also received regular oral paracetamol (Pharmacare PSM Healthcare Ltd, Auckland, New Zealand 1 gm every 6 hours, regular diclofenac (Voltaren, Novartis, New Zealand) 75 mg bd and oral tramadol (50–100 mg) or immediate release oral morphine (Douglas Pharmaceutical, Auckland, New Zealand) 10–20 mg orally as required. Patient pain was assessed daily using a visual analogue scale from 1–10 with 10 being severe pain and a score of 1, no pain.

Patients also received intravenous antiemetic therapy with droperidol (Droleptin, AFT Pharmaceuticals Ltd, Auckland, New Zealand) 0.5 mg, dexamethasone (Douglas Pharmaceuticals, Auckland, New Zealand) 8 mg, ondansetron (Pacific Pharmaceuticals, Auckland, New Zealand) 4 mg, and metoclopromide (Pacific Pharmaceuticals Ltd, Auckland, New Zealand) 10 mg at induction as prophylaxis against postoperative nausea and vomiting (PONV) syndrome.

General endotracheal anesthesia was induced with intravenous propofol (Dipravan, Fresofol Astra Zeneca Pty Ltd, North Ryde, New South Wales, Australia) and maintained with desflurane in air (Suprane, Baxter Healthcare, Old Toongabbie, New South Wales, Australia) and oxygen. Neuromuscular blockade was produced with atracurium 0.5 mg/kg (Glaxo Smith Kline, New Zealand). Fentanyl was also administered at a dose of 3–5 *μ*g/kg intravenously. All patients had their tracheas extubated at the end of the procedure and no patient was managed with elective postoperative positive pressure ventilation.

All patients were managed intraoperatively with sequential calf compression devices, urinary catheters, and arterial lines. Most patients were also managed with central venous cannulae, although 8 of the patients managed with intrathecal morphine did not undergo central venous cannulation and were managed with peripheral venous lines only as they were undergoing minor liver resections of a single segment or less. No patients received nasogastric tube insertion intraoperatively. Surgical drains were not placed routinely after hepatectomy but nonsuction drains were placed in patients who had undergone a biliary reconstruction. Postoperatively all patients spent the first night in surgical intensive care before being transferred to the surgical ward on postoperation day 1.

### 2.4. Surgical Technique

The surgical technique employed was identical in all cases. Hepatectomy was undertaken through a right subcostal incision with a midline extension. In lobar or segmental resections inflow structures and venous drainage were taken sequentially prior to parenchymal division. Parenchymal division was undertaken in all cases using the harmonic scalpel (Ethicon Endosurgery, Johnson and Johnson, Auckland, New Zealand). Large parenchymal structures were controlled with ligaclips or silk ligatures. In patients requiring nonanatomical resection of subcapsular liver lesions, tumors were elevated using a 3-0 non-absorbable suture and the harmonic scalpel was used to divide surrounding parenchyma. Large parenchymal structures were controlled with ligaclips or silk ligatures. Inflow occlusion (Pringle maneuver) was not used routinely and utilized only when deemed necessary to control ongoing blood loss during parenchymal division (12 patients in the epidural analgesia group and 11 patients in the intrathecal morphine group). When utilized, inflow occlusion was in periods of 5 minutes with 3–5 minutes recovery. No ischemic preconditioning was employed. Patients managed with intrathecal morphine also received wound infiltration with 30 mL 0.75% ropivicaine (Naropin, AstraZeneca Pty Ltd, North Ryde, NSW 2113, Australia).

All incisions were closed with an absorbable, subcuticular suture, and a waterproof dressing applied. This dressing is transparent and allowed assessment of the underlying incision. It remained in place and was changed on postoperative day 3.

### 2.5. Postoperative Nursing Care Plan

Immediately following surgery, patients spent approximately 60 minutes in a specialized recovery area adjacent to the operating theatres and were then transferred to a high dependency unit. On day one postoperation patients were transferred to a general surgical ward. All patients were managed with a standard postoperative nursing care plan (Table 1). This was introduced in 2003 and specified routine daily management, such as frequency of physiological observations (pulse, blood pressure, respiratory rate, and oxygen saturation), daily dietary orders and, mobilization orders. All vascular access lines were removed on postoperative day 1 apart from the central venous line which was used to take blood daily to check full blood count, creatinine and electrolytes, liver function tests, and INR. In patients managed with epidural analgesia the central venous line was used to maintain intravenous fluids until the epidural was weaned. The urinary catheter was also left in situ in these patients until postoperative day 3 (Table 2). 

### 2.6. Statistical Analysis

Data is expressed as the median and range except where specified. Intergroup comparisons were performed using an analysis of variance. A *P* ≤ .05 was considered to be statistically significant. 

## 3. Results

### 3.1. Demographics and Patient Details

The patient characteristics and diagnoses were similar in both the intrathecal morphine and epidural groups (Table 3) as were the types of hepatic resection and the numbers of patients treated with chemotherapy prior to resection (Table 4). The median duration of surgery was 180 minutes (range 70–300 minutes) and 190 minutes (range 65–270 minutes) in the intrathecal morphine and epidural groups (*P* = .78). The median blood loss in the epidural group was 352 ± 73 mL (SEM; range 150–3200 mL) and 277 ± 88 mL (SEM; range 170–2800 mL) in the intrathecal morphine group (*P* = .57).

Abdominal drains were used in only 2 patients (1 each from the epidural and intrathecal morphine groups) who underwent resections for hilar cholangiocarcinomas and required biliary reconstructions.

### 3.2. Fluid Balance

Patients managed with intrathecal morphine received less intraoperative intravenous fluids mean 1458 mL ± 245 mL(range 1200–2800 mL) versus 1920 mL ± 490 mL (range 1500–3200 mL; *P* = .06), less postoperative fluids mean 1019 mL ± 227 mL (range 800–1500 mL) versus mean 3840 mL ± 377 mL (range 3500–5200 mL; *P* = .03). Intravenous dopamine was used to support blood pressure in 24 of 50 patients treated with epidural anesthesia in the first 12 hours post liver resection. In line with institutional policy intravenous dopamine was weaned in all patients prior to their transfer to the ward from the postoperative intensive care. In 38 of 50 patients managed with intrathecal morphine, IV fluids were discontinued on day 1 with the remaining 12 patients receiving maintenance fluids for postoperative day 1 before they were discontinued on day 2. In comparison all 50 patients managed with epidural anesthesia required maintenance IV fluids on postoperative days 1–3 inclusive. Of these patients, 22 required additional intravenous fluid boluses (in 500 mL aliquots of crystalloid) postoperatively to maintain satisfactory urine output and blood pressures. Twenty eight of 50 patients in the epidural group received postoperative diuretic therapy with frusemide and spironolactone on postoperative days 5–15 to mobilize retained fluid.

**Figure 1:**
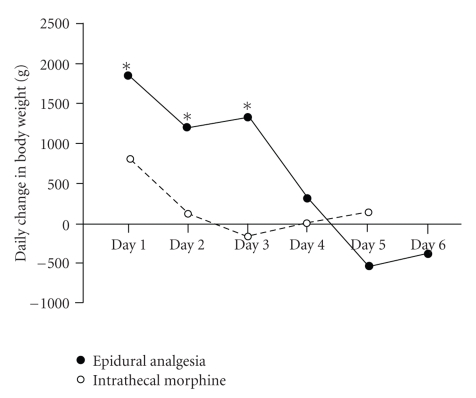
Median daily change in patient in grams in the postoperative period in patient patients managed with intrathecal morphine or epidural anesthesia (*P < .05 with respect to intrathecal morphine group).

Analysis of patients daily weights demonstrate a significant postoperative gain in the epidural group in comparison to the intrathecal morphine group (Figure 1) reflecting the effect of significant fluid loading and sequestration in these patients.

### 3.3. Analgesic Requirement and Pain Scores

No complications were recorded following administration of intrathecal morphine or placement of epidural catheters.

Median daily pain scores are presented in Table 5. Analgesia was equivalent between both the intrathecal morphine and epidural analgesia groups on postoperative days 1 and 2. However patients managed with epidural anesthesia reported significantly higher pain scores overall after weaning of the epidurals on postoperative day 3. Pain scores were again equivalent on postoperative days 4 and 5 (Table 5).

**Figure 2:**
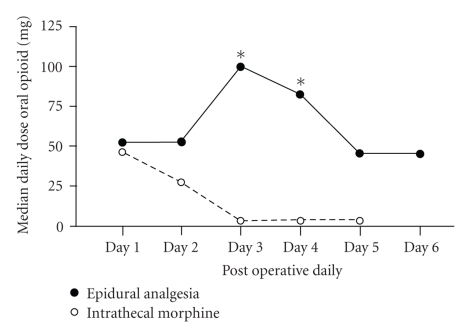
Median daily requirement of oral opiate (mg) in the postoperative period in patients managed with intrathecal morphine or epidural anesthesia (*P < .05 with respect to intrathecal morphine group).

All patients received regular oral paracetamol (1000 mg Q6hrly orally per day). However patients in the epidural group were more likely to require additional oral opiate given as either tramadol or oral morphine particularly on postoperative day 3 and 4 (Figure 2).

### 3.4. Physiological Function

All patients treated with intrathecal morphine were recorded as having passed flatus on postoperative day 1. In the epidural group, 12 are recorded as passing flatus on postoperative day 1, 28 on postoperative day 2, and 10 on postoperative day 3. All patients in the intrathecal morphine group resumed a regular diet at a median of 16 hours post operation (breakfast on postoperation day 1) and maintained a normal diet for the duration of their admission. Patients in the epidural group took longer to establish on a normal diet at 20 hours postoperation (lunch on postoperative day 1; *P* = .05). Subjectively these patients had greater difficulty maintaining regular food intake particularly around day 3 postoperation when their epidurals were discontinued and an oral dose of morphine was administered.

All patients treated with intrathecal morphine mobilized fully and independently around the surgical ward by the end of postoperative day 1. In comparison patients managed with epidural analgesia mobilized around their room on postoperative day one and only 16 mobilized fully around the surgical ward before their epidural were removed on postoperative day 3. The reasons for this reduced rate of mobilization were light headedness and dizziness related to an upright position, difficulty mobilizing with an infusion pump, urinary catheter and intravenous fluids running, and perceived reduced leg strength.

### 3.5. Outcome and Hospital Stay

Complication related data is summarized in Table 6 and the results for patients undergoing major hepatectomy are summarized in Table 7. There were no perioperative deaths in either groups. Overall 16% of patients in the intrathecal morphine group and 22% (*P* = .68) in the epidural group developed complications. All complications were managed non-operatively. One patient from the intrathecal morphine group was readmitted for radiological drainage of a parahepatic biloma (5-day-readmission, total hospital stay 10 days) and 2 patients from the epidural group were readmitted (1 patient for intravenous antibiotics for a wound infection—4 day readmission, total hospital stay 12 days—and 1 patient for percutaneous drainage of a biloma—6 day readmission, total hospital stay 13 days). The total hospital stay (including readmissions) was 4.7 ± 0.9 days for the intrathecal morphine group versus 6.8 ± 1.2 days for the epidural group (*P* = .02).

## 4. Discussion

This investigation describes the use of single dose intrathecal morphine for perioperative analgesia in comparison to continuous epidural infusion in patients undergoing hepatic resection. Prior to 2005 epidural anesthesia was utilized in all patients undergoing hepatic resection although we had begun to develop a comprehensive package of care for these patients emphasizing standardized management protocols, early resumption of regular diet and early mobilization. The routine use of intrathecal morphine has enhanced our postoperative care in that it has enabled hepatic resection to be undertaken with minimal adverse effects on patients normal physiology and functioning.

Patients treated with intrathecal morphine mobilized and resumed a normal diet earlier than patients managed with epidural catheters. There are a number of reasons for this. Firstly the majority of these patients did not require maintenance intravenous fluid therapy beyond postoperative day 1 and urinary catheters could be removed on transfer from the surgical high dependency unit making it physically easier to mobilize without multiple encumbrances. Secondly, patients treated with epidural catheters often complained of postural related light headedness and perceived lower limb weakness making it difficult for them to mobilize. Epidural catheters were removed on postoperative day 3 in all cases but this was often accompanied by a period of less optimal pain relief and nausea following the administration of oral morphine. Consequently these patients often mobilized poorly on postoperative day 3. 

Both patient groups were able to tolerate a regular diet by the end of postoperative day 1, however, the group managed with intrathecal morphine consistently established normal dietary intake before the group managed with epidural catheters. While epidural analgesia does block the effects of sympathetic activation on the gastrointestinal tract reducing the likelihood of ileus, it is accompanied by the requirement for significant intravenous fluid loading to counteract the cardiovascular effects of sympathetic blockade. Patients with epidurals required significantly more intravenous fluid on postoperative days 1 to 3 resulting in significant weight gain. Lobo et al. [5] have shown that fluid administered in this way is primarily sequestered in the intracellular compartment and is accompanied by a delay in the return of gastrointestinal function. We believe that the decreased requirement for intravenous fluids in the intrathecal morphine treated patients contributed significantly to their rapid mobilization, return of gastrointestinal function, and reduced hospital stay. In addition, the doses of oral morphine administered following removal of the epidural catheters on postoperative day 3 were accompanied by significant nausea and often retarded optimal food intake at this time in the postoperative course. In comparison intrathecal morphine was associated with very good analgesia and consistently low pain scores throughout the hospital admission but acted only centrally meaning that, in these patients, the gastrointestinal tract, was not exposed to the adverse effects of opioids.

Both intrathecal morphine administration and epidural analgesia were safe and there were no adverse effects recorded from their administration. In both patient groups intrathecal and epidural access was undertaken in the context of a normal coagulation profile. Epidural catheters were removed once coagulation has normalized after hepatic resection and with-holding routine aniti-thromboembolic therapy. Epidural or intrathecal hematoma are theoretical complications of intrathecal morphine therapy and have been documented [6, 7]. Centrally mediated respiratory depression is also a recognized adverse event [8]. No patient in this series required administration of naloxone for this indication. However the risk of respiratory depression is significant in the first 12 to 16 hours postoperation and for this reason these patients do need to be observed in high dependency unit or an intensive care unit [9]. In addition, intrathecal morphine should be used with caution in elderly patients (>80 years of age), patients with chronic respiratory disease and obstructive sleep apnoea. We found that patients respiratory rates would often fall to 8–10 per minute in recovery but they consistently recorded oxygen saturations of >97%, were easily rousable and denied pain. This may reflect the influence of gabapentin which has anxiolytic properties and induces dissociation [10]. 

The risk of respiratory depression with intrathecal morphine is dose related. The initial description of this technique employed a single dose of 500 *μ*g of morphine for hepatic resection [9]. In comparison doses of 100–150 *μ*g are used for patients undergoing lower limb surgery with minimal adverse respiratory effects [11]. The dose of 300 *μ*g used in this investigation was a deliberate attempt to administer an adequate intrathecal blockade to permit hepatic resection without increasing the frequency of adverse events. In addition all patients treated with intrathecal morphine were managed and monitored in a surgical intensive for at least 12 hours following hepatectomy. While this technique does shorten overall hospital stay it does not reduce the intensity of care required to monitor for signs of respiratory depression or hemorrhage in the immediate postoperative period.

While this investigation has focused on the positive effects of intrathecal morphine in comparison to epidural analgesia. It is important to emphasize that this occurred as part of an overall package of care. This care plan has been developed and refined over 5 years and the patients in the current investigation were managed between 2005 and 2007. While the choice of postoperative analgesia was the major difference between the groups, all patients benefited from a comprehensive care package that was improved over time. Patients were fully informed of the standard operative course and educated in the importance of early mobilization and respiratory care. Nursing staff were also proactive in mobilizing patients treated with either intrathecal morphine or epidural analgesia and normalizing their care as soon as appropriate. Hepatic resection was also undertaken with care to avoid significant and sudden hemorrhage and other complications such as air embolism. Pringle inflow was not routinely used to protect and optimize liver remnant function and surgical drains were only employed in patients undergoing biliary reconstruction [12]. It is worth emphasizing that intrathecal morphine may not be so successful in other forms of intra-abdominal surgery in comparison to hepatic resection. In this series, access to the liver was via a right upper quadrant incision. Laparotomy was performed to exclude the presence of extrahepatic disease but the small bowel was not excessively handled and remained in an intra-abdominal position for the duration of the procedure. Undoubtedly this contributed to the lack of significant ileus in our patients and making the oral route available for early administration of medications and nutrition. Surgery accompanied by significant small bowel handling and manipulation may mean that it is harder to normalize postoperative medication and dietary requirements.

This technique has important implications for hepatic surgery. Laparoscopic hepatectomy is currently under intensive investigation and hospital stays of 5 days are reported following major laparoscopic resections and used to justify its introduction [13]. However questions remain regarding the safety and applicability of laparoscopic hepatectomy. Many series report conversion rates of 8%–15% for intraoperative hemorrhage and margin positivity rates of 2% or greater for resections for cancer [14]. While laparoscopic surgery does reduce the morbidity due to large upper abdominal incisions, careful application of a number surgical and anesthetic techniques to open surgery can also make it possible to substantially reduce hospital stay following open surgery.

## Figures and Tables

**Table 1:** Nursing care plan for patients managed with intrathecal morphine.

Day of Surgery	Admit to surgical ward
	Normal medications continued (excluding anticoagulants)
	Nil by mouth 4 hours before surgery
	Below knee TED stockings fitted
	Liquid/light diet *ad libertum* on waking

Postoperative Day 1	Check full blood count, electrolytes, liver function, INR
	Remove arterial line and calf compression device
	Luer central venous line
	Remove urinary catheter
	Unrestricted diet *ad libertum *
	Normal medications
	Mobilize as able

Postoperative Day 2	Check full blood count, electrolytes, liver function, INR
	Unrestricted diet *ad libertum *
	Mobilize as able

Postoperative Day 3	Check full blood count, electrolytes, liver function, INR
	First surgical dressing change
	Unrestricted diet *ad libertum *
	Mobilize as able

Postoperative Day 4	Check full blood count, electrolytes, liver function, INR
	Unrestricted diet *ad libertum *
	Mobilize as able
	Review discharge criteria

Postoperative Day 5	Check full blood count, electrolytes, liver function, INR
	Unrestricted diet *ad libertum *
	Remove central venous line
	Discharge

**Table 2:** Nursing care plan for patients managed with continuous epidural analgesia.

Day of Surgery	Admit to surgical ward
	Normal medications continued (excluding anticoagulants)
	Nil by mouth 4 hours before surgery
	Below knee TED stockings fitted
	Liquid/light diet *ad libertum* on waking
	Vital signs monitoring postoperatively including hourly respiratory rate for 24 hours

Postoperative Day 1	Check full blood count, electrolytes, liver function, INR
	Remove arterial line and calf compression device
	Central venous line maintenance fluids
	Unrestricted diet *ad libertum *
	Normal medications
	Mobilize as able

Postoperative Day 2	Check full blood count, electrolytes, liver function, INR
	Central venous line maintenance fluids
	Unrestricted diet *ad libertum *
	Mobilize as able

Postoperative Day 3	Check full blood count, electrolytes, liver function, INR
	Central venous line maintenance fluids
	First surgical dressing change
	Stop epidural and remove catheter
	Remove urinary catheter
	Unrestricted diet *ad libertum *
	Mobilize as able

Postoperative Day 4	Check full blood count, electrolytes, liver function, INR
	Unrestricted diet *ad libertum *
	Mobilize as able

Postoperative Day 5	Check full blood count, electrolytes, liver function, INR
	Unrestricted diet *ad libertum *
	Remove central venous line
	Mobilize as able

Postoperative Day 6	Unrestricted diet *ad libertum *
	Remove central venous line
	Mobilize as able
	Review discharge criteria

Postoperative Day 7	Unrestricted diet *ad libertum *
	Remove central venous line
	Mobilize as able
	Discharge

**Table 3:** Patient demographics and diagnoses (HCC = hepatocellular carcinoma).

	Intrathecal Morphine (*n* = 50)	Epidural Analgesia (*n* = 50)	Significance
Median age (range)	60 yrs (23–79)	61 yrs (28–83)	0.87
Sex ratio (M : F)	24 : 26	27 : 23	0.56
ASA Grade			
I	11	13	0.47
II	32	31	0.76
III	7	6	0.89
Pre-op Chemotherapy	27	25	0.73
Parenchymal Quality			
Normal	40	39	0.89
Steatosis	8	9	0.83
Cirrhosis	2	2	0.97
Diagnosis			
Colorectal Mets	31	32	0.88
Neuroendocrine Mets	6	5	0.89
HCC	2	3	0.77
Cholangiocarcinoma	3	1	0.79
Benign Tumours	8	9	0.88

**Table 4:** Details of operative procedures.

	Intrathecal Morphine (*n* = 50)	Epidural Analgesia (*n* = 50)	Significance
Hemihepatectomy plus ≥1 metastatectomies	6	8	0.78
Hemihepatectomy	12	11	0.94
Extended hemihepatectomy	2	2	0.97
Multiple segmentectomy (≥2 segments)	9	10	0.87
Monosegmentectomy	10	12	0.88
Metastatectomy	11	7	0.64
Pringle time	12 patients 5 min 45 sec (range 3 min 30 sec to 12 min 58 sec)	11 patients 5 min 17 sec (range 3 min to 9 min 17 sec)	0.59

**Table 5:** Median daily postoperative pain scores in patients treated with intrathecal morphine or epidural analgesia.

	Intrathecal Morphine (*n* = 50)	Epidural Analgesia (*n* = 50)	Significance
Postoperative Pain Score			
Day 1	1	2	0.78
Day 2	0	1	0.67
Day 3	0	4	0.05
Day 4	1	2	0.64
Day 5	1	1	0.93
Day 6	—	1	—

**Table 6:** Summary of postoperative complications.

	Intrathecal morphine (*n* = 50)	Epidural analgesia (*n* = 50)	*P*
Patients with complications	8 (16%)	11 (22%)	.59
Biloma	1 (2%)	1 (2%)	.89
Hepatic insufficiency	2 (4%)	3 (6%)	.88
Wound infection	3 (6%)	4 (8%)	.73
Pleural effusion	1 (2%)	2 (4%)	.78
Pneumonia	—	1 (2%)	—
Urinary tract infection	1 (2%)	—	—

**Table 7:** Overall comparison between intrathecal morphine and epidural analgesia groups in patients undergoing major (≥4 segments) liver resection.

	Intrathecal morphine (*n* = 50)	Epidural analgesia (*n* = 50)	*P*
Major Liver Resection	20	21	—
Duration of Surgery	178 ± 34 min	184 ± 32 min	.67
Pringle time	6* *min ± 87 sec	5 min ± 69 sec	.39
Operative Blood Loss	352 mL ± 69 mL	312 ± 78 mL	.66
Passage Flatus Day 1	19 patients	6 patients	.03
Regular Diet Day 1	20 patients	6 patients	.05
Full Mobilization Day 1	20 patients	0	.01
Complication Rate	1 patient	2 patients	.39
Hospital Stay	4.6 ± 0.8 days	7.2 ± 1.3 days	.05
